# Effect of shape of titanium dioxide nanofillers on the properties of dental composites

**DOI:** 10.1007/s10266-023-00784-2

**Published:** 2023-01-12

**Authors:** Prajna P. Nayak, Sudarshan Kini, Kishore Ginjupalli, Deepika Pai

**Affiliations:** 1 Department of Pedodontics and Preventive Dentistry, Manipal College of Dental Sciences, Manipal Academy of Higher Education, Manipal, Karnataka 576104 India; 2 Department of Pedodontics and Preventive Dentistry, Nitte (Deemed to Be University), AB Shetty Memorial Institute of Dental Sciences (ABSMIDS), Deralakatte, Mangalore, Karnataka 575018 India; 3 Nitte (Deemed to Be Univerisity), Nitte University Centre for Science Education and Research, Paneer Campus, Deralakatte, Mangalore, Karnataka 575018 India; 4 Department of Dental Materials, Manipal College of Dental Sciences, Manipal Academy of Higher Education, Manipal, Karnataka 576104 India

**Keywords:** Dental composites, Flexural strength, Nano filler morphology, Shear bond strength, Titanium dioxide

## Abstract

The main objective of the present study was to evaluate the effect of the morphology of titanium dioxide nanofillers on the flexural strength and shear bond strength of the dental composite. Spherical and rhombic-shaped nano titanium dioxide fillers were synthesized via solvothermal method and were characterized. Subsequently, these fillers were incorporated into a flowable composite (Filtek^™^ Z350 XT Flowable Restorative) at 0.5 wt.% and 1.5 wt.% and the prepared specimens were stored in water for 24 h. The specimens were then evaluated for flexural strength using a universal testing machine. Similarly, the shear bond strength of modified composites to the tooth was evaluated and bond failures were analyzed using stereomicroscope magnification. Incorporation of nanofillers significantly enhanced the flexural strength of flowable composite (*p* = 0.009) with a significant increase at 0.5wt.% of spherical (*p* = 0.015) and rhomboidal-shaped fillers (*p* = 0.010). However, no statistically significant difference in flexural strength was observed among the different shapes of nanofillers. The results of our study did not show a significant effect on the shear bond strength of the composites. Thus the reinforcing ability of titanium dioxide nanofillers on dental composite was confirmed in this study, although the effect of using nanofillers with different morphology was not significant.

**Graphical abstract:**

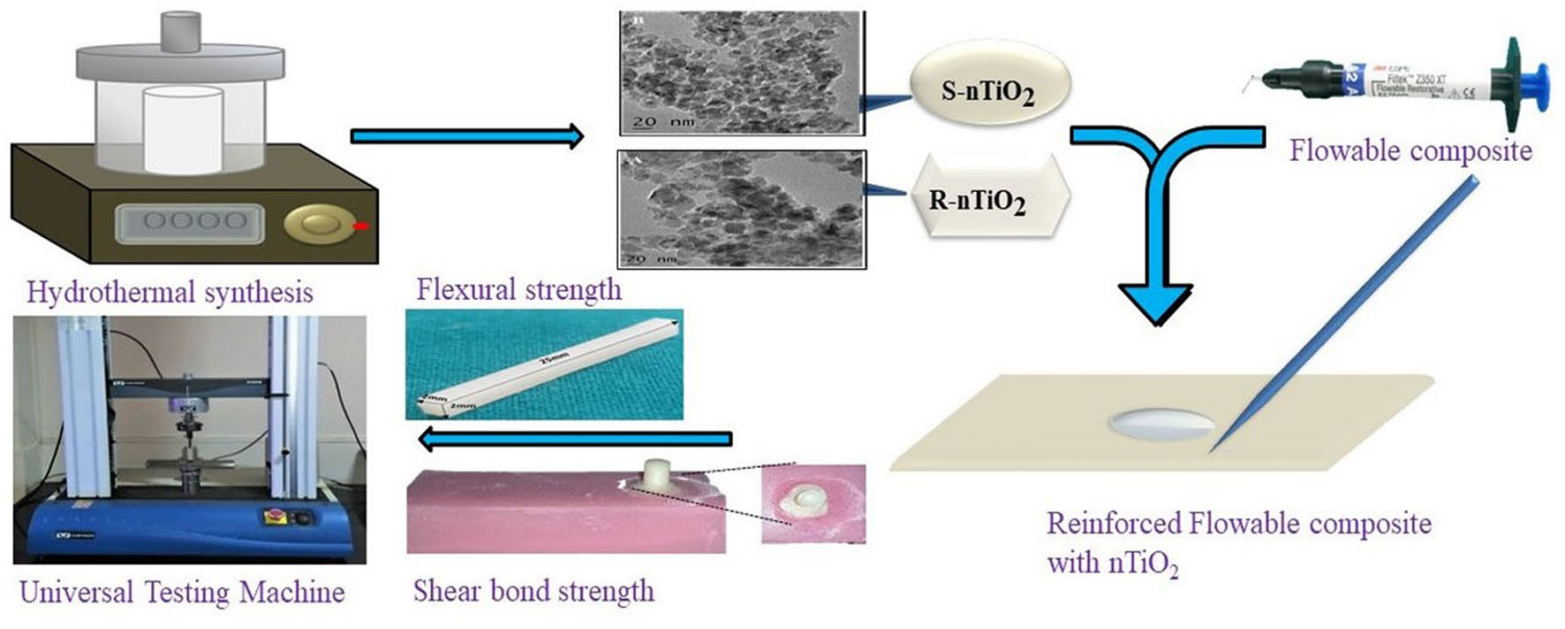

## Introduction

Growing awareness and concerns regarding amalgam toxicity with demand for aesthetics from patients have led to the increased use of tooth-coloured restorations. In paediatric dentistry a child patient’s cooperating ability is one of the factors considered for the selection of the restorative technique. Glass ionomer cements with superior handling properties have been the material of choice to date. However, glass ionomer cements are more prone to occlusal wear [1] and mechanical strength of the same does not reach par with amalgam. Composite resins on the other hand, in spite of being technique sensitive are proven to exhibit superior mechanical and aesthetic properties. A recent systematic review on the survival of restorations in primary teeth showed the lowest annual failure rate for composite restorations (1.7–12.9%) [2]. In permanent teeth, composite resins have been the first material of choice for direct restorations with an annual failure rate ranging from 1 to 3% [3, 4]. Further advancements in dental composites in terms of filler size, shape and volume have resulted in the introduction of conventional/traditional, microfilled, small particle filled, and hybrid composites. The main driving force behind such developments was to improve the mechanical properties of dental composites without compromising the esthetic qualities.

Mechanical stability is a critical factor for the long-term success of resin composites used in dentistry [5, 6]. Restorations involving multiple surfaces, predominantly Class II and Class IV restorations, are prone to fractures due to excessive concentration of stresses at the proximal region and incisal edges [4, 7–9]. According to recent systematic reviews, fracture is one of the common causes of composite restoration failure in the posterior region [4, 5, 10].

A direct correlation between the filler characteristics and mechanical properties of dental composite resins is well acknowledged. Hence, most strategies aiming to improve the mechanical characteristics of dental composites revolve around the development of novel filler materials and altering their size, shape and concentration in the resin matrix. It has been reported that both filler size and morphology directly influence the mechanical properties of resultant composites [11–13]. Though colloidal silica and glasses with heavy metals are most commonly employed, fibers made of glass and ceramics have also been investigated to reinforce dental restorative materials [14–17]. However, some of these attempts did not fructify owing to the large filler size, uneven filler distribution and weak bonding of filler to the resin matrix [14, 18].

With the advent of nanotechnology, various nanosized fillers in dental composites have been extensively investigated. Nanofillers exhibit unique physical and chemical properties, including a high surface area to volume ratio for better interaction with the resin matrix [19]. As the size of nanofillers approaches the wavelength of visible light, it allows easy passage of the light through the resin matrix compared to microfillers that reflect the light, thus providing flexibility to adjust the shade of the composite. Further, their smaller size facilitates easy polishability with a superior surface finish. Therefore, the incorporation of nanofillers offers a promising approach to enhance both the esthetic and mechanical properties of dental restorative materials [14, 18].

Titanium dioxide has been used as a filler in various dental restorative materials owing to its influence on colour, superior strength, corrosion resistance and biocompatibility. Incorporation of titanium dioxide nanoparticles into the resin composites is known to increase the opalescence and at concentrations between 0.1 and 0.25% mimic the opalescence of human enamel [20]. In addition, the nanoparticulate form of titanium dioxide has been reported to exhibit antimicrobial activity [3]. Several studies have demonstrated the ability of nano-titanium dioxide to improve the mechanical properties of dental materials [21, 22]. A significant increase in fracture toughness, flexural strength and flexural modulus was observed in dental composites reinforced with titanium dioxide [14]. Similarly, the compressive strength of glass ionomer cements significantly increased with the incorporation of 3% of titanium dioxide nanoparticles [23].

Despite intense research on the suitability of various materials as fillers in dental composites, to the best of our knowledge, the effect of morphology of fillers, nano-sized fillers in particular, on the mechanical characteristics of dental composites has not been reported widely. Therefore, the present study aims to evaluate the influence of the incorporation of various shapes and concentrations of titanium dioxide nanoparticles on the flexural and shear bond strength of the flowable composite. The null hypothesis of the study was that the shape/morphology and concentration of titanium dioxide nano-fillers does not enhance the flexural strength and shear bond strength of the flowable resin composites.

## Materials and methods

The details of the materials used in the study are presented in Table 1

**Table 1:** Materials used in the present study

Materials	Details
Titanium (IV) butoxide	CAS No. 5593-70-4, Sigma Aldrich, USA
Oleic acid	CAS No. 112-80-7, Sigma Aldrich, USA
Oleylamine	CAS no. STBG8339, Sigma Aldrich, USA
Absolute ethanol	CAS no. 200-578-6, Hayman grade, UK
Iso-propanol	CAS no. 67-63-0, AR Grade, Merck USA
Flowable composite resin	Filtek^™^ Z350 XT Flowable Restorative 3 M ESPE, St Paul MN, USA
Visible light cure composite curing unit	Elipar^™^ 3 M ESPE, St Paul MN USA
Phosphoric acid etchant	Scotchbond^™^, 3 M ESPE, St Paul MN, USA
Bonding agent	Adper^™^ Single Bond 2 TM, 3 M ESPE, St.Paul, MN, USA
Self-cure poly methyl methacrylate resin	Rapid Repair, Dentsply Pvt. Ltd, India
Diamond disk	Ortho Classic, USA
Vinyl polysiloxane impression material	Reprosil, Dentsply Pvt. Ltd, USA

### Synthesis of titanium dioxide nanoparticles of different shapes via solvothermal method

Anisotropic titanium dioxide nanoparticles were prepared via the solvothermal method as reported earlier with few modifications [24]. Five mM of titanium butoxide (TB) was added to a mixture of oleic acid (OA) and oleylamine (OM) in different ratios in a Teflon-lined stainless steel autoclave (Techinstro, India) containing isopropanol (100 mM). The ratio of TB:OA:OM for the synthesis of spherical and rhombic-shaped nanoparticles was 1:6:4 and 1:5:5, respectively. This mixture was stirred for 10 min and transferred into an autoclave containing an azeotropic mixture of 96% iso-propanol and water (20 ml). The autoclave was heated at 180 °C in a sand bath for 18 h. The whitish-yellow precipitate so obtained was centrifuged and washed several times with propanol and dried in a vacuum desiccator. The dried powder was annealed at 500 °C for 20 h and stored in a desiccator [24].

### Characterization of titanium dioxide nanoparticles

#### X-ray diffraction (XRD)

The crystalline structure of TiO_2_ nanoparticles was analysed using the X-ray diffraction method. X-ray diffraction patterns with CuKα radiation (*λ* = 0.154 nm) were investigated using a Rigaku Ultima-IV Powder X-Ray Diffractometer. Braggs equation was used to evaluate the lattice spacing of nanocrystals represented as *d* = *nλ*/(2 sin *θ*_B_), where *λ* is X-ray wavelength (CuKα = 0.154 nm), n is an integer, and *θ*_B_ is the half scattering angle of the diffraction peaks on 2*θ* scale.

#### Fourier transform infrared spectroscopy

Fourier transform infrared spectroscopy (FT-IR) (Jasco FT/IR6200 spectrometer, Japan) was used to determine the functional groups present on the surface of the nanoparticles. The FTIR spectra were recorded at room temperature using the transmission mode, covering the spectral range from 450 to 4500 cm^−1^. A pure KBr pellet was used with and without synthesized titanium dioxide nanoparticles and its IR spectrum was recorded.

#### High-resolution transmission electron microscopy (HR-TEM)

High-resolution transmission electron microscopy (Jeol/JEM 2100, Japan) measurements were carried out at 200 kV. HR-TEM images were analysed using Image J software and the size distribution profile was plotted as a histogram. The corresponding selected area electron diffraction (SAED) of HR-TEM samples was analysed to confirm the diffraction of the titanium dioxide phase.

#### Preparation of composites with titanium dioxide nanoparticles

A commercially available flowable composite was incorporated with titanium dioxide nanoparticles of different morphology in varying amounts. Initially, a preweighed amount of flowable composite was dispensed onto a paper pad and an appropriate amount (0.5 or 1.5% by weight) of spherical and rhombic nanoparticles were weighed and incorporated into the composite by thoroughly mixing the composite and nanoparticles using a plastic spatula. Flowable composite groups containing spherical titanium dioxide nanofillers at 0.5 wt.% (F-St-O.5) 1.5 wt.% (F-St-1.5), and containing rhombic titanium dioxide nanofillers at 0.5 wt.% (F-Rt-0.5) and 1.5 wt.% (F-Rt-1.5) were tested for mechanical properties. Commercial flowable composite without nanofiller was considered as control.

### Evaluation of mechanical properties of nanocomposites

#### Flexural strength

Flexural strength samples were prepared in accordance with ISO 4049/2019 standard [25]. Flowable composite with or without titanium dioxide nanoparticles was filled into an aluminium split mold of 25 × 2 × 2 mm covered with mylar strip on top and bottom. Subsequently, the composite was cured using an LED curing unit with an intensity of 1200 wM/cm^2^ for 30 s on both sides of the specimen. Entire length of the sample was cured in 3 segments for 30 s in consideration of the opaque metallic mold and the diameter of the light guide of the curing unit which was about 8 mm in diameter. The cured specimens were retrieved and stored in water for 24 h before testing. Flexural strength measurement was carried out using a three-point bending method (*n* = 10). The samples were supported at both ends by placing them on a flexural jig on the lower platform of the universal testing machine (Model 3366, Instron Corporation, United Kingdom) and were loaded at a crosshead speed of 0.5 mm/min until fracture. The flexural strength was calculated by dividing the maximum load recorded during the test by the area of the sample using the formula $\sigma = \frac{3FL}{{2bd^{2} }}$ where *F* stands for force, *L* is the length of the specimen, *b* is the breadth of the specimen*,* and* d* is the thickness of the specimen.

#### Measurement of shear bond strength

About fifty healthy human premolar teeth extracted for orthodontic purposes were collected and cleaned for calculus and debris by scaling. The extracted teeth were then stored in 0.5% chloramine-T at 4 °C for 7 days for disinfection and transferred to distilled water for one day before use. Distilled water used was not changed and was maintained at 37 °C temperature. The teeth were sectioned horizontally with a diamond disk beneath the dentinoenamel junction to expose the coronal dentin surface. The exposed tooth surfaces were finished and polished using 400 and 600-grit carbide polishing paper. The teeth were then mounted onto custom-made molds (50 × 20 × 15 mm) of auto-polymerizing pink orthodontic resin, with a coronal portion of the tooth exposed. The exposed dentin of the tooth was treated with Scotchbond^™^ 3 M ESPE etchant for 15 s, rinsed with water for 10 s and dried under a gentle air stream for 10 s. This was followed by the application of two layers of bonding agent Adper^™^ Single bond two. The bonding agent was uniformly distributed using a gentle air stream and light-cured for 20 s (Elipar^™^ 3 M ESPE).

A plastic tube (Tygon) with an internal diameter of 3 mm and a height of 4 mm was placed on the treated dentin surface. Subsequently, tygon was filled with flowable composite in 2 mm thickness and light-cured for 20 s. Subsequently, tygon was removed from the composite cylinder and the samples were stored in deionized water for 24 h before testing. Shear bond strength was measured by applying a compressive force perpendicular to the composite specimen on the tooth using Universal Testing Machine (Model 3366, Instron Corporation, United Kingdom) at a crosshead speed of 0.5 mm/min until debonding. Shear bond strength was calculated by dividing the maximum load recorded during the test by the area of the sample (*n* = 10).

After shear bond strength measurement, debonded surfaces of the teeth were observed under a stereomicroscope at 25× magnification and the failure patterns were classified as either Cohesive (fracture occurred within dental composite), Adhesive failure (debonding occurred at the interface between the tooth and dental composite) or Mixed (a combination of adhesive and cohesive failure observed on the tooth).

### Statistical analysis

The data followed normal distribution which was confirmed by Kolmogorov–Smirnov test. The overall comparison of mean values of flexural strength and shear bond strength was carried out by ANOVA followed by TUKEY Post hoc test. The type of bond failures was statistically compared using chi-square test.

## Results

### Characterization of titanium dioxide nanoparticles

**Fig. 1:**
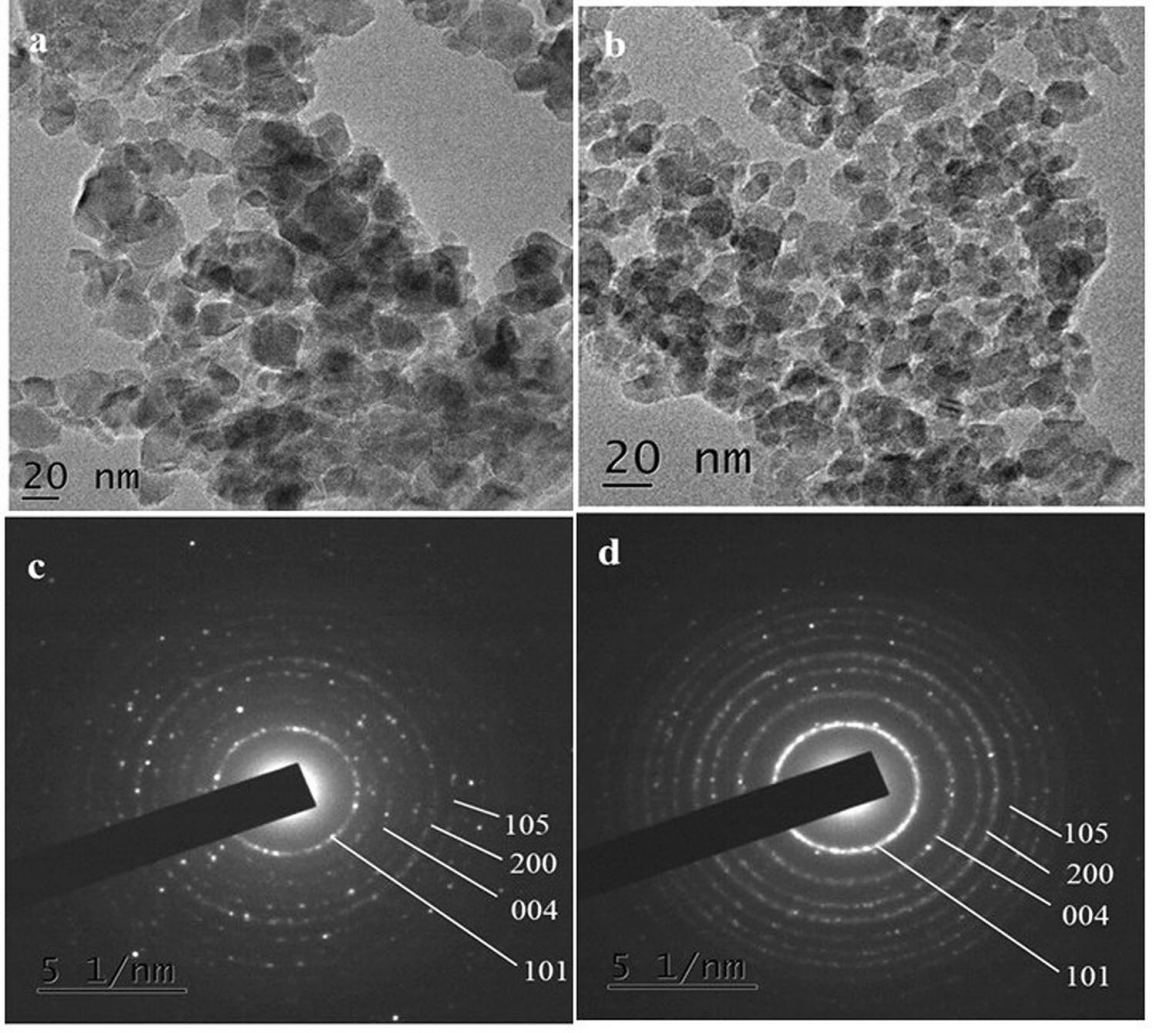
HR-TEM and SAED images of TiO_2_ nanoparticles. **a** and **c** are HR-TEM and SAED images of Rhombic titanium dioxide nanoparticles; **b** and **d** are HR-TEM and SAED images of Spherical titanium dioxide nanoparticles

HR-TEM images (Fig. 1a and b) show that synthesized titanium dioxide nanoparticles exhibit spherical and rhombic shapes. TiO_2_ nanoparticles prepared using TB:OA:OM ratio of 1:6:4 exhibited a spherical shape with an average particle size of 35 ± 4 nm. In contrast, TB:OA:OM ratio of 1:5:5 favoured the formation of rhombic-shaped nanoparticles with an average particle size of 12 ± 4 nm.

**Fig. 2:**
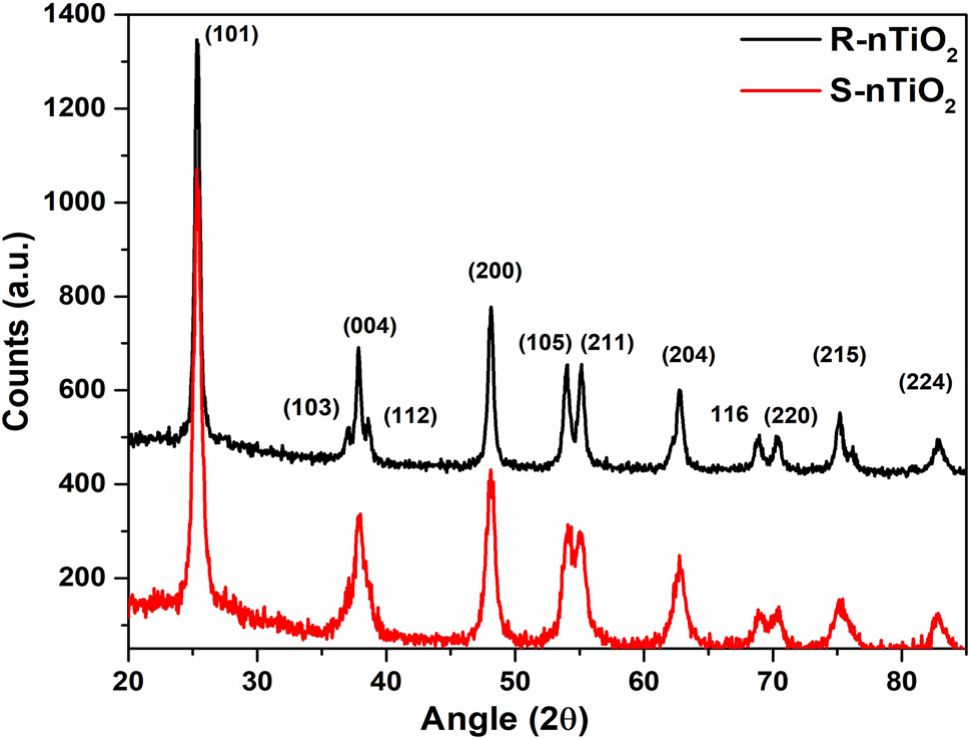
X-ray diffraction spectra of TiO_2_ nanoparticles of different shapes

SAED analysis (Fig. 1c and d) and X-ray diffraction of annealed powder sample revealed a pure anatase phase for nanocrystalline TiO_2_ synthesized in the study. XRD analysis of the powders revealed crystalline planes (101), (004), (200), (105), (211), (204), (116), (220), (215) and (224) corresponding to pure anatase phase for both TiO_2_ nanoparticle samples (JCPDS File No. 21-1272). In addition to these, Rhombic TiO_2_ nanoparticles showed growth of nanocrystals along (103) and (112) directions (Fig. 2), indicating the formation of anisotropic TiO_2_ nanoparticles as reported previously [24]. Crystalline size of XRD peaks was determined using the Debye–Scherrer formula (*D* = *Kλ*/β cos *θ*) and was found to be 18 and 12 nm for spherical and Rhombic TiO_2_ nanoparticles, respectively.

**Fig. 3:**
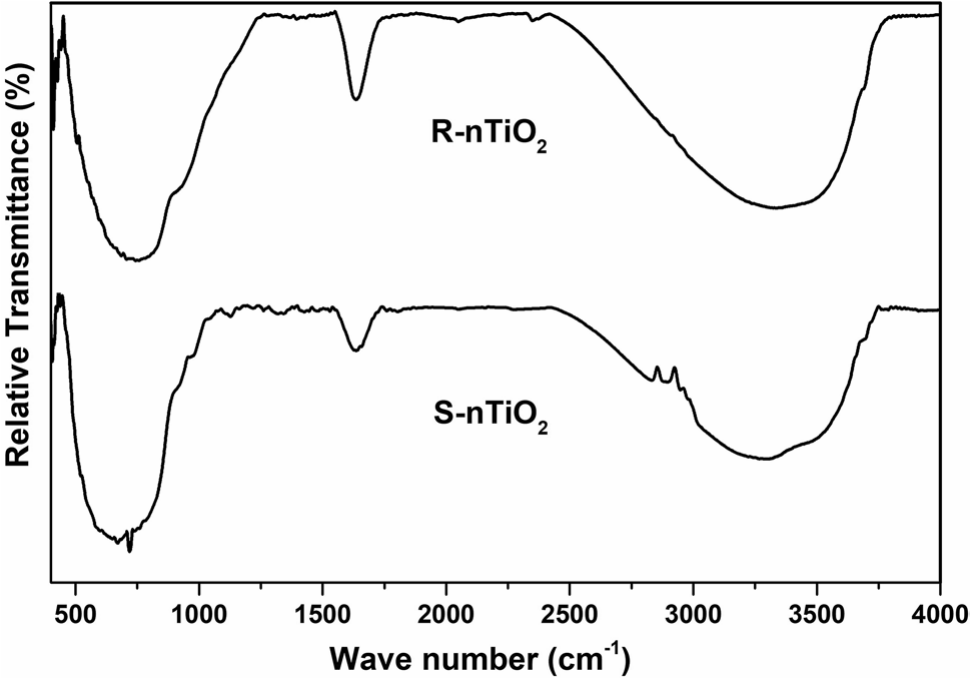
FTIR spectra of TiO_2_ nanoparticles of different shapes

FTIR spectrum of TiO_2_ powder (Fig. 3) showed samples with a broad peak around 425–1200 cm^−1^ attributed to the stretching vibrations of Ti–O groups whereas broad peaks observed at 3350 and 1630 cm^−1^ correspond to the surface adsorbed water and hydroxyl groups.

### Evaluation of mechanical properties

#### Flexural strength

**Fig. 4:**
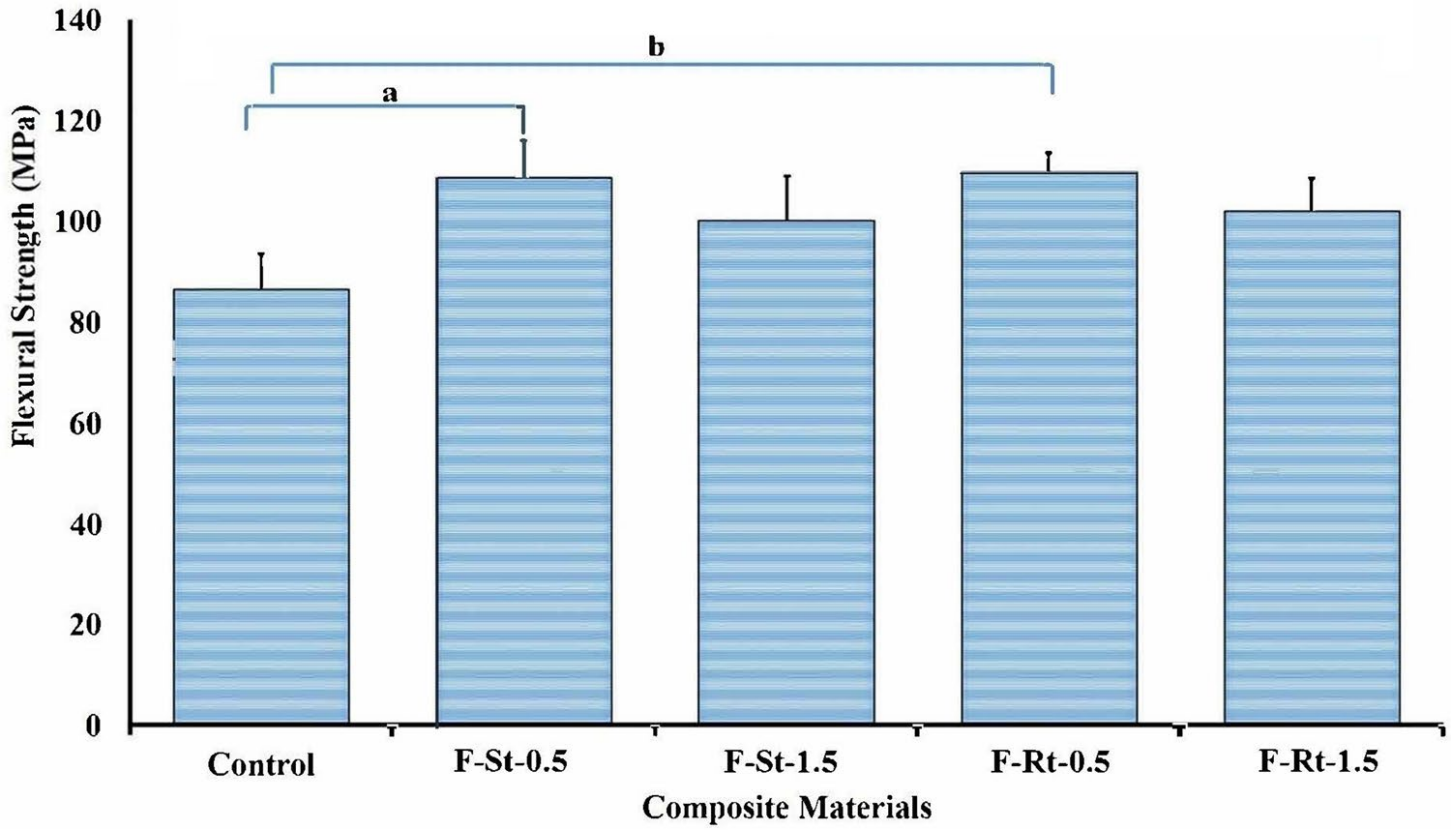
Flexural strength (mean ± SD) of flowable composite incorporated with TiO_2_ nanoparticles of different shapes. ANOVA analysis showing statistical significance has been represented as **a** *p* ≤ 0.05, **b** *p* ≤ 0.01

The effect of nanofillers on the flexural strength of flowable composites is presented in Fig. 4. One-way ANOVA was used to analyse the flexural strength and the means of all the experimental groups was significantly higher than the control (*p* = 0.009). Pairwise post hoc analysis was conducted using Tukey’s method to compare groups and a significant increase in flexural strength was seen in group F-St-0.5 (*p* = 0.015) and F-Rt-0.5 (*p* = 0.010) in comparison to the control. However, the comparisons based on the shape and weight percentage of the nanofillers showed no significant difference in the flexural strength as presented in Table 2.

**Table 2:** Post Hoc analysis for flexural strength using Tukey test

Post Hoc analysis using the Tukey test							
Dependent variable	Material (*I*)	Material (*J*)	Mean difference (*I*–*J*)	Std. error	Sig	95% confidence interval	
						Lower bound	Upper bound
Flexural strength	Control	F-St-0.5	– 22.28400^*^	6.74537	0.015	– 41.4506	– 3.1174
		F-St-1.5	– 13.74300	6.74537	0.265	– 32.9096	5.4236
		F-Rt-0.5	– 23.28500^*^	6.74537	0.010	– 42.4516	– 4.1184
		F-Rt-1.5	– 15.62300	6.74537	0.159	– 34.7896	3.5436

#### Shear bond strength

**Fig. 5:**
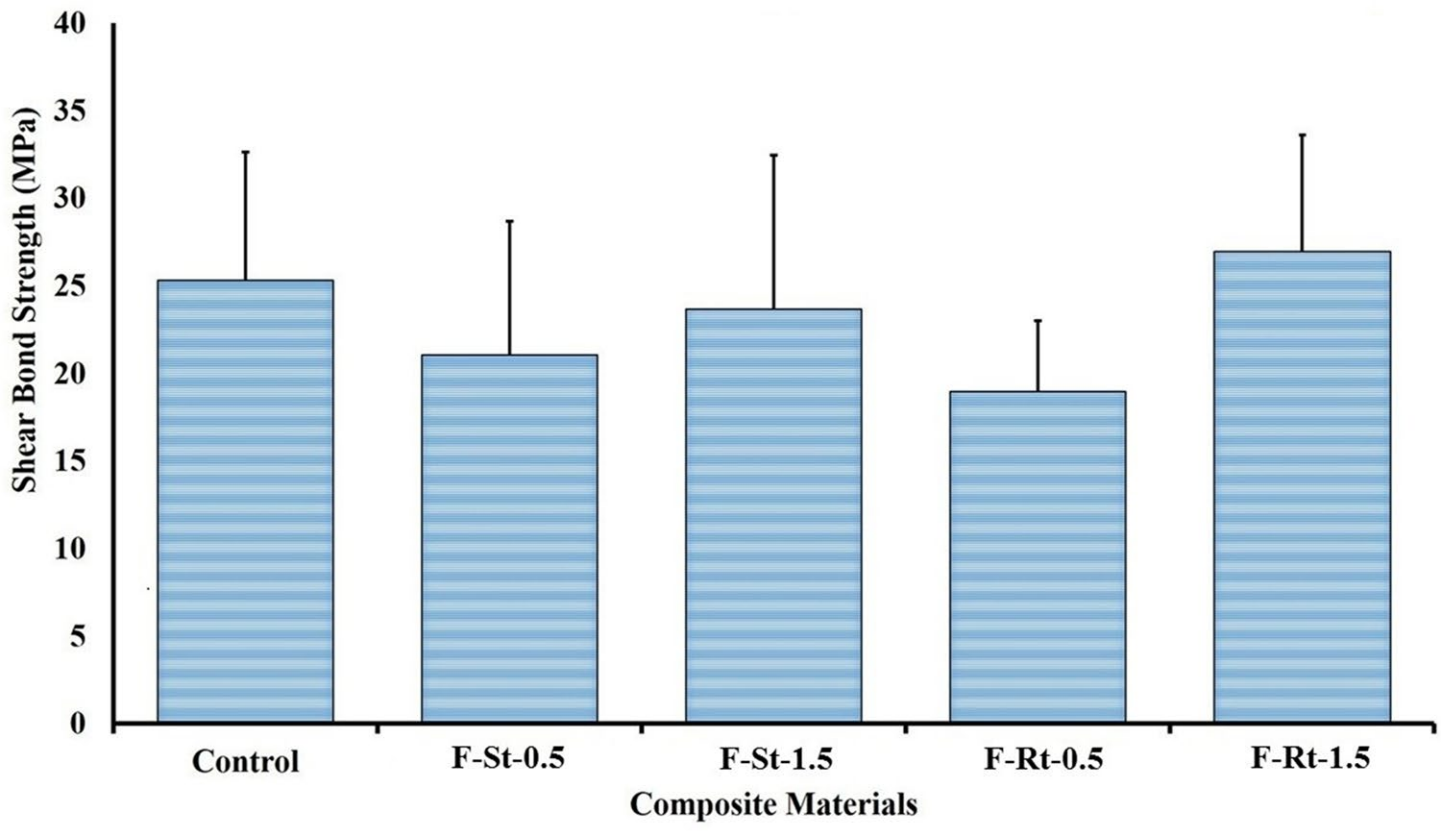
Shear bond strength (mean ± SD) of flowable composite incorporated with TiO_2_ nanoparticles of different shapes to the tooth structure

The effect of nanofillers on the shear bond strength of flowable composites to the tooth structure is presented in Fig. 5. One-way ANOVA was used to analyse the means of all the experimental groups and the results indicated that the incorporation of morphologically different nanoparticles in the flowable composite did not significantly alter the shear bond strength in comparison to control (*p* = 0.092).

#### Modes of bond failures

**Fig. 6:**
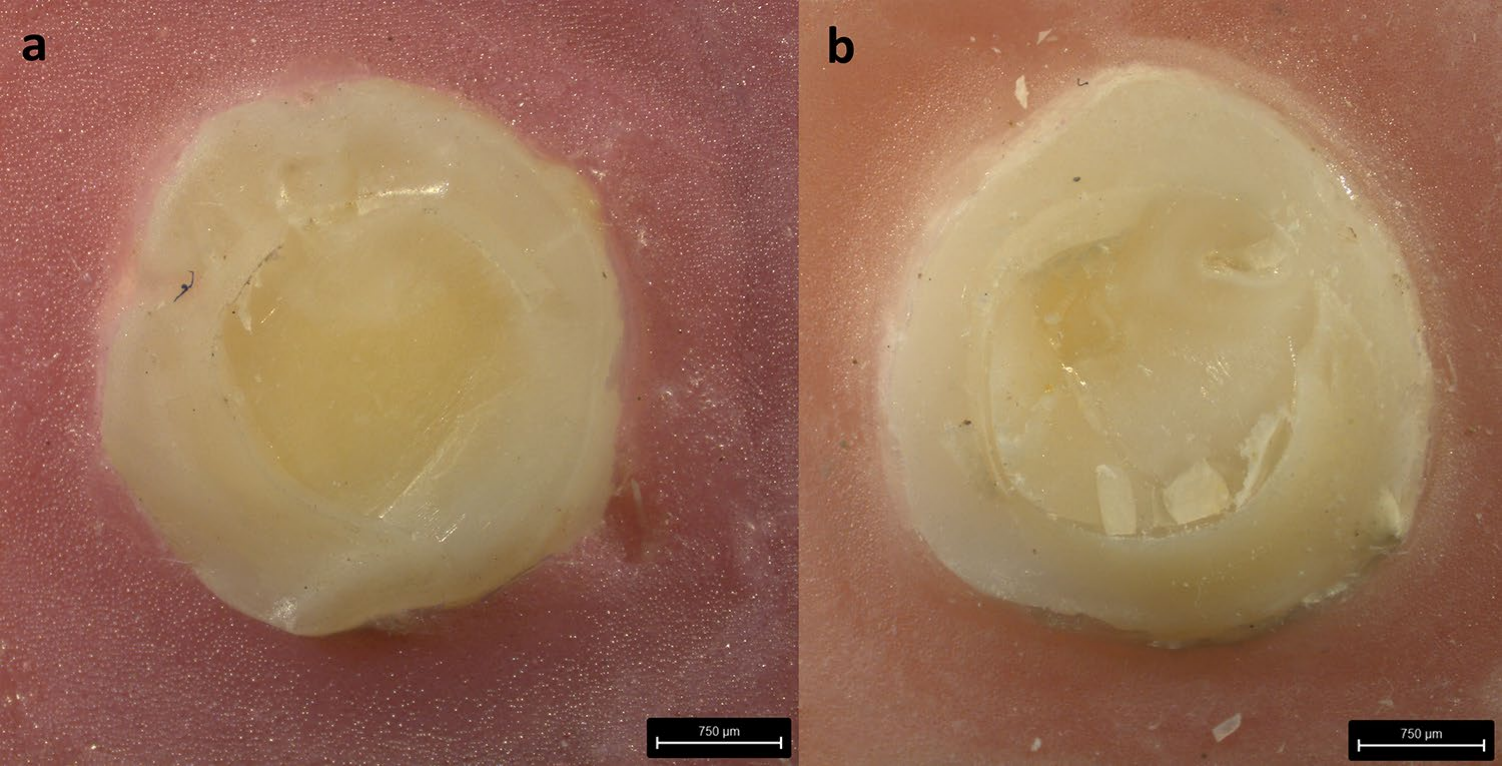
Modes of bond failures assessed using stereomicroscope magnification at 25× . **a** samples similar were assessed as adhesive failures; **b** samples with part of bonded flowable composite retained onto the teeth were assessed as a mixed failure

**Fig. 7:**
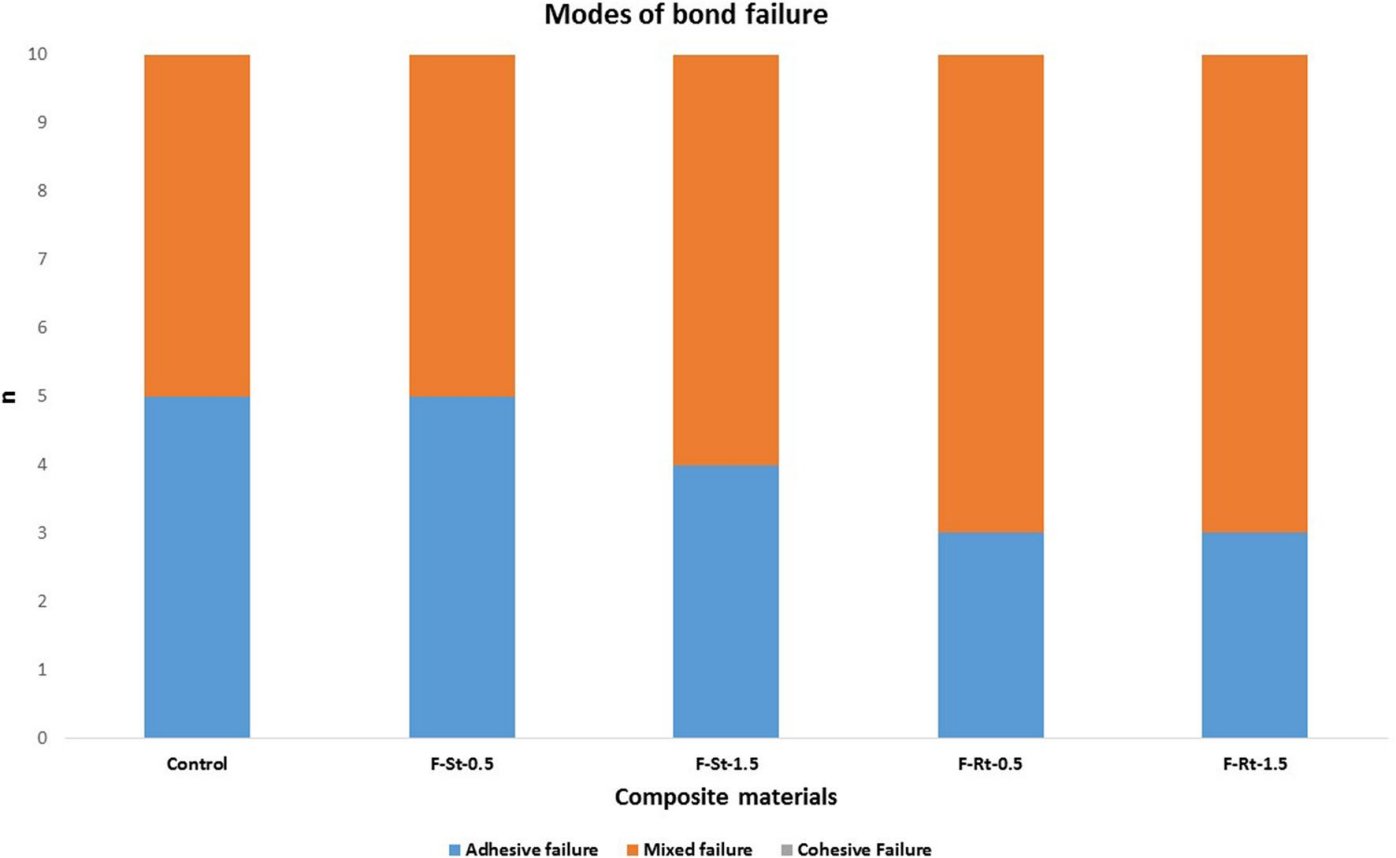
Column chart displaying different failure modes observed in flowable composite incorporated with TiO_2_

The modes of bond failures were assessed using stereomicroscope magnification are presented in Fig. 6. The results were analysed using Chi-square test. Control and F-St -0.5 groups showed an equal number of mixed (*n* = 5) and adhesive (*n* = 5) failures. The remaining groups showed a higher number of mixed failures with no cohesive failure within the tooth or composite (Fig. 7). The results were analysed using Chi-square test and statistically, no significant differences in the failure modes were observed among the groups (*p* = 0.872).

## Discussion

The main aim of the present study was to investigate the effect of variations in filler morphology on their reinforcing ability in dental composites. An ideal resin composite is expected to possess low polymerization shrinkage, superior mechanical properties, excellent handling characteristics, and aesthetics [26]. According to a meta-analysis, at least 5% of posterior resin restorations failed due to fracture and 10% of them showed excessive wear within ten year period of observation [9, 27] and the replacement rate was higher after seven to ten years [28].

Fillers in the resin composites are stronger than the resin matrix, thereby directly influences the mechanical properties and their size has been widely used for their classification in dental applications [21, 28–30]. Filler particle size, size distribution, loading, hardness, index of refraction and radiopacity play an important role in their selection. Composites with large filler particles exhibit superior mechanical strength but poor aesthetics as they are difficult to polish due to the selective wear of the softer resin matrix leading to protrusion or plucking of fillers. Composites with smaller filler particles, in contrast, are easy to polish but exhibit inferior mechanical characteristics as such fillers exhibit large surface area and hence require large amounts of resin to wet their surface [21, 28–30]. Hence, small filler sizes with wide size distribution are generally preferred to improve the mechanical properties of composites without compromising on their polishability.

Recently, a variety of nanomaterials such as titanium dioxide, silica, hydroxyapatite, silver, zinc oxide and graphene oxide have been evaluated as fillers to reinforce resin composites used in dentistry to enhance mechanical properties and impart antibacterial properties [22, 30–33]. However, some of the nanomaterials, such as silver nanoparticles and graphene nanoparticles, are known to cause discoloration and thereby compromise aesthetics [34]. Among them, titanium dioxide has been widely used in dentistry as an opacifying agent. It exhibits excellent biocompatibility and mechanical properties [14, 35].

The pure anatase form of TiO_2_ nanoparticles is prepared by a solvothermal process employing hydrolysis of titanium alkoxides in the presence of surface stabilizers under non-oxidizing conditions at relatively low temperatures. The pure crystalline anatase phase was obtained in white powder by calcination at 400–500 °C [24, 36, 37]. Controlling hydrolytic reaction is challenging and can lead to a diverse population of different shapes of nanoparticles. Using long carbon chain hydrophobic surfactants such as oleic acid and oleylamine in optimum ratio, monodispersed rhombic, bar, spherical, truncated rhombic, dog-bone shaped nanoparticles have been synthesized previously [23]. Adding a trace amount of water with surface stabilizers in a non-aqueous solvent yield shape-controlled and highly crystalline nanoparticles, which may be suitable for use in dental applications [24].

In the present study, the shapes of nanoparticles were controlled by varying the ratio of TB:OA:OM, as confirmed by HR-TEM. The capping agent OA binds strongly on the surface of TiO_2_ via its free carboxyl groups, whereas OM has been shown to support the growth of rhombic-shaped nanoparticles [24, 38]. Further, the slow hydrolysis rate of TB facilitated the controlled morphological distribution of nanoparticles. Therefore, the ratio of TB:OA:OM was found to be a critical factor in the synthesis of anisotropic TiO_2_ nanoparticles. Compared to spherical-shaped nanoparticles, the crystallinity of TiO_2_ was more prominent in the rhombic group, suggesting that the optimum ratio of TB:OA:OB to synthesize uniform crystalline TiO_2_ nanoparticles is 1:5:5 at 180 °C. SAED and XRD analysis confirmed the pure anatase phase, as indicated by different crystal planes observed in both samples. However, crystalline growth along (103) and (112) plane direction was only observed in nanoparticles causing rhombic anisotropy when the ratio of stabilizing agents was 1:5:5. The crystalline size data further supported the anisotropic nanoparticle formation. FTIR study indicated Ti–O bonding thus justifying TiO_2_ nanoparticle formation.

Flowable composites are used widely in pediatric dental practice with indications in areas requiring improved mechanical strength, such as minimally invasive class II lesions, fracture reattachment cases, preventive resin restorations, as cavity liners, splinting of traumatized teeth, for bonding of fiber posts in endodontically treated teeth, etc. Flowable composites are referred to as an inhomogeneous group of materials with a wide range of applicability. Thus modification of this group of the composite provides the clinician a prospect to choose the right material for the indicated clinical situation. Thus the addition of titanium oxide nanofillers can alleviate the mechanical properties of flowable composite to facilitate its use in areas demanding better mechanical properties [35, 39, 40]. Earlier study by Darfur et al. showed a significant increase in the elastic modulus and fracture toughness of the flowable composites when reinforced with titanium nanotubes. The enhancement of mechanical properties was evident at all concentrations (0–5%), however, a minimal decrease in flowability and radiopacity was observed at concentrations above 3% of filler reinforcement. Therefore, in our study 0.5 wt.% and 1.5 wt.% of TiO_2_ were used to reinforce the flowable composite and significant increase in flexural strength was observed [35].

Addition of nTiO_2_ increased the flexural strength of the flowable composite compared to the control group. A similar improvement in strength was observed in earlier investigations [14, 35]. It indicates that TiO_2_ nanoparticles reinforced the matrix of flowable composite leading to better flexural strength. However, a reduction in flexural strength with increasing concentration of nTiO_2_ could be attributed to the agglomeration of nanoparticles resulting in poor adhesion between resin matrix and nanoparticles [14, 41, 42]. Alternatively, these nanofillers can be functionalized to overcome their agglomeration and to facilitate uniform dispersion in the resin matrix [35, 43–46]. The improvement in mechanical characteristics of flowable composites was found to be similar irrespective of morphologies of nTiO_2_. Nevertheless, further comparison with other anisotropic nanoparticles is necessary to justify the effect of the anisotropy of nanofillers on the mechanical properties of dental composites.

Shear bond strength test was used to evaluate the adhesion between the teeth and reinforced flowable composites. Ease and speed, with the lack of specimen processing requirements, make it the most common and reliable technique to test bonding [47]. The results depicted that the nanoparticle shape and amount did not alter the shear bond strength of flowable composites. Though an increase in shear bond strength was evident with increasing concentration of TiO_2_ nanoparticles, no significant difference in shear bond strength between the reinforced flowable composite and the tooth was observed. A similar insignificant increase in shear bond strength with glass ionomer reinforced with TiO_2_ nanoparticles was reported [21].

Analysis of failure modes revealed that debonding between the tooth and reinforced composite occurred through mixed failure indicating firm bonding of the composite to the tooth during failure [20, 29]. However, there was no statistically significant difference in the mode of bond failures between the groups.

On the basis of results obtained in the present study, the null hypothesis could only be accepted partially as the incorporation of TiO_2_ nanoparticles significantly increased the flexural strength of the flowable composite although no significant changes in the shear bond strength to natural tooth was observed. The limitations are that the study compares only two morphologies of the fillers and silane coupling of the fillers is not performed. Further studies with different anisotropic forms and functionalisation of fillers are recommended to confirm the effect of the shape of fillers on the mechanical properties and to recommend a specific shape of the filler that demonstrates better mechanical properties for dental applications.

## Conclusions

The present study concludes that the reinforcing ability of two different morphologies of TiO_2_ nanoparticles evaluated was not significantly different. Incorporation of TiO_2_ nanofillers significantly enhanced the flexural strength of flowable composites compared to control irrespective of their shape suggesting that TiO_2_ nanofillers affect the flexural strength of flowable composites. However, there is no significant difference in flexural strength for the different weight percentage of TiO_2_-incorporated composite samples. Similarly, TiO_2_ nanofiller incorporation did not affect the shear bond strength of the flowable composites and no significant differences in the bond failure modes is observed among the groups.

Nevertheless, these results form a basis and provide scope for further investigations on the effect of nanoparticles of different shapes and a combination of different shapes along with their particle size distributions in enhancing the mechanical properties of dental composites.

## Data Availability

The datasets generated during and /or analysed in this study are available from the correspoding author on reasonable request.
